# Supplementary material for: Pneumocystis jirovecii pneumonia mortality risk associated with preceding long-term steroid use for the underlying disease: A multicenter, retrospective cohort study
Source: PLoS One. 2024 Feb 8;19(2):e0292507. doi: 10.1371/journal.pone.0292507 (PMC10852346; doi:10.1371/journal.pone.0292507)
Supplement: Attachment — Submitted filename: Response to Reviewers.docx [file pone.0292507.s003.docx]

Response to Reviewers

**Reviewer #1: line 120, there are no reliable methods to differentiate PCP infection from colonization. Given high sensitivity of PCR, it is possible that there are pneumocysits infected patients without pulmonary symptoms but positive for PCR alone. More important, this exclusion made the authors miss some important cases. The authors are encouraged to re-word this portion.**

Response: We agree with the reviewer. We have removed the colonization detection part from the relevant section and added a discussion part about the PCP diagnostic dilemma.

**Fig. 2, X axis should be "1-Specificity"**

Response: I have corrected the relevant part.

**Fig.3, there are no signs of statistical analysis.**

Response: We have added a description of the statistical analysis for the relevant part in the section.

**Line 331-334, the authors are encouraged to extend to discuss the dilemma and challenges of PCP diagnosis based on the following references.**

**Liu B, Totten M, Nematollahi S, Datta K, Memon W, Marimuthu S, Wolf LA, Carroll KC, Zhang SX. Development and Evaluation of a Fully Automated Molecular Assay Targeting the Mitochondrial Small Subunit rRNA Gene for the Detection of Pneumocystis jirovecii in Bronchoalveolar Lavage Fluid Specimens. J Mol Diagn. 2020 Dec;22(12):1482-1493. doi: 10.1016/j.jmoldx.2020.10.003. Epub 2020 Oct 15. Erratum in: J Mol Diagn. 2021 Apr;23(4):506. PMID: 33069878.**

Recponse: We appreciate your comment and the reference you suggested. We have revised the limitation section to acknowledge that many cases may be overlooked due to the sensitivity issue of PCR.

**changes to the reference list**

We have made changes because the publication issue has been decided.

(15. Miyake K, Kawamura T, Nakahara Y, Sasaki S. A single-center, person-month-based analysis of the risk of developing Pneumocystis pneumonia (PCP) in immunosuppressed non-HIV patients: Preventive effects of trimethoprim-sulfamethoxazole. ) J Infect Chemother. 2023;S1341-321X(23)00174-5. doi: 10.1016/j.jiac.2023.07.012. to J Infect Chemother. 2023; 29: 1097-1102.

We have added references.

59. Liu B, Totten M, Nematollahi S, Datta K, Memon W, Marimuthu S, Wolf LA, et al. Development and Evaluation of a Fully Automated Molecular Assay Targeting the Mitochondrial Small Subunit rRNA Gene for the Detection of Pneumocystis jirovecii in Bronchoalveolar Lavage Fluid Specimens. J Mol Diagn. 2020;22:1482-1493.
